# Serial Monitoring of Optic Nerve Sheath Diameter for Prediction of Functional Outcome in Acute Ischaemic Stroke: A Diagnostic Accuracy Study

**DOI:** 10.7759/cureus.89659

**Published:** 2025-08-08

**Authors:** Silpa S, Poonam Arora, Bharat Bhardwaj, Rajnish Arora, Nilotpal Chowdhury, Udit Chauhan, Takshak Shankar, Sreejith Jayachandran, Aishwarya Nair, Hari Prasad

**Affiliations:** 1 Emergency Medicine, All India Institute of Medical Sciences, New Delhi, New Delhi, IND; 2 Emergency Medicine, All India Institute of Medical Sciences, Rishikesh, Rishikesh, IND; 3 Neurosurgery, All India Institute of Medical Sciences, Rishikesh, Rishikesh, IND; 4 Pathology, All India Institute of Medical Sciences, Rishikesh, Rishikesh, IND; 5 Radiology, All India Institute of Medical Sciences, Rishikesh, Rishikesh, IND

**Keywords:** ischemic stroke, modified rankin scale score, optic nerve sheath diameter, stroke, ultrasound

## Abstract

Background

Increased intracranial pressure (ICP) can be reliably detected at the bedside using the optic nerve sheath diameter (ONSD). The functional outcome in stroke patients can be predicted with the use of acute-phase ONSD dynamics.

Objectives

To determine the predictive accuracy of ONSD on days 0, one, and three for the prognosis of ischemic stroke patients presented to emergency medicine as measured by Modified Rankin Scale (mRS) score.

Methods

The study involved the enrollment of patients who presented to the emergency department with clinical and radiological diagnosis of ischemic stroke within 24 hours of onset of symptoms. On the day of the incident, day one and day three, the optic nerve sheath diameter was measured transorbitally, 3 mm behind the optic disc. On days 28 and 90, the patient’s functional outcome was evaluated using the mRS score. An mRS score of ≤ 3 is considered a good functional outcome, and > 3 is considered a poor functional outcome.

Results

Study participants were sixty-six people who had experienced an acute ischemic stroke. Mean age was 55.30 ± 13.99, and 56.1% of patients were male. The mean ONSD at all time points during serial monitoring differed significantly between the poor and good functional outcomes. The change in ONSD over time, i.e., from day 0 to day three, was statistically significant in poor functional outcome (Friedman Test: χ2 = 25.6, p < 0.001) for day 28, as well as day 90 (Friedman Test: χ2 = 27.3, p < 0.001). In good functional outcome patients, the change in ONSD for the above-mentioned period was not statistically significant on day 28, as well as on day 90. Cut off ONSD 0.47 cm on day 0 can predict poor functional outcome with 100% specificity and 61% sensitivity for day 28 and 73% sensitivity for day 90.

Conclusion

Measurement of ONSD with ultrasound had a moderate potential to predict poor functional outcome (mRS > 3) on day 90 in individuals presenting to the emergency room. The trend of ONSD on days 0 to three can assist in predicting functional prognosis in acute ischemic stroke.

## Introduction

Stroke is the second leading cause of death globally [1]. In 2017, the global stroke burden was estimated to be 11.9 million cases and 6.1 million fatalities [2]. Stroke is a prominent cause of disability, particularly in the elderly population, where stroke incidence is highest [3].

The rate of mortality due to haemorrhagic stroke is more than the ischemic type [4]. The reason for poor prognosis and long hospital stay in haemorrhagic stroke is due to more increase in intracranial pressure (ICP) [5]. Cerebral edema is the major cause of rapid deterioration and mortality in stroke victims. Ischemic stroke accounts for the majority, while haemorrhagic accounts for 10-15% of all strokes [6]. So, measurement of ICP in ischemic stroke patients can help to predict functional outcome and take timely actions in the majority of stroke individuals. ICP can be measured at the bedside non-invasively using optic nerve sheath diameter (ONSD) sonography. Moreover, it has moderate accuracy in differentiating haemorrhagic from ischemic stroke [7]. Many studies have observed the role of ONSD in haemorrhagic stroke; unstable trends of ONSD performed at admission, on the second, and the third day are associated with worse outcomes in these patients [8]. We aimed to evaluate the prognostic utility of serial ONSD measurements for predicting functional outcomes in patients with acute ischemic stroke.

In acute ischaemic stroke, cerebral edema starts to form in the first 24 to 48 hours after the stroke and peaks three to five days later [9,10]. So, there can be a role of measuring ONSD on the day three of ischemic stroke, therefore, we intend to assess role of serial ONSD on days 0, one and three in the prognosis of ischemic stroke who presented to emergency medicine, to analyse the difference of ONSD from baseline and to investigate the relationship between functional outcome and ONSD.

Objectives of the study

This study was conducted with the following specific objectives:

1. To evaluate whether optic nerve sheath diameter (ONSD) measurements at different time points (days 0, one, and three) can predict functional outcomes in patients with acute ischemic stroke.

2. To assess the diagnostic performance of ONSD values using receiver operating characteristic (ROC) analysis for poor neurological outcomes (mRS > 3) at 28 and 90 days.

3. To explore the potential role of serial ONSD monitoring as a non-invasive tool for early detection of elevated intracranial pressure and poor prognostic trajectory.

## Materials and methods

Study and design

This prospective cohort study was carried out from November 2021 to April 2023 in a tertiary care facility.

Selection of patients

Adult (> 18 years) patients who presented with a clinical and/or radiological diagnosis of ischaemic stroke within 24 hours of symptom onset were included. This study comprised patients who had an ischaemic stroke as determined by imaging. Individuals excluded from the study are: Diagnosis of hemorrhagic stroke on initial neuroimaging, presence of other non-stroke causes of focal neurological deficit (e.g., seizure, hypoglycemia, mass lesions), mechanical ventilation at the time of ONSD measurement, prior history of intracranial space-occupying lesions (ICSOL) or prior brain surgery, anatomical abnormalities or injuries at the ONSD measurement site (e.g., traumatic globe injury, enucleation, or evisceration), known diagnosis of idiopathic intracranial hypertension, or recent head trauma within the past six weeks. 

Sample size

The sample size was calculated to have a power of 90% at an alpha of 0.05 and an expected area under the ROC curve of 0.7. So, the estimated sample size for our study was 66 ischemic stroke patients [11].

Measurement of optic nerve sheath diameter using ocular ultrasonography

**Figure 1:**
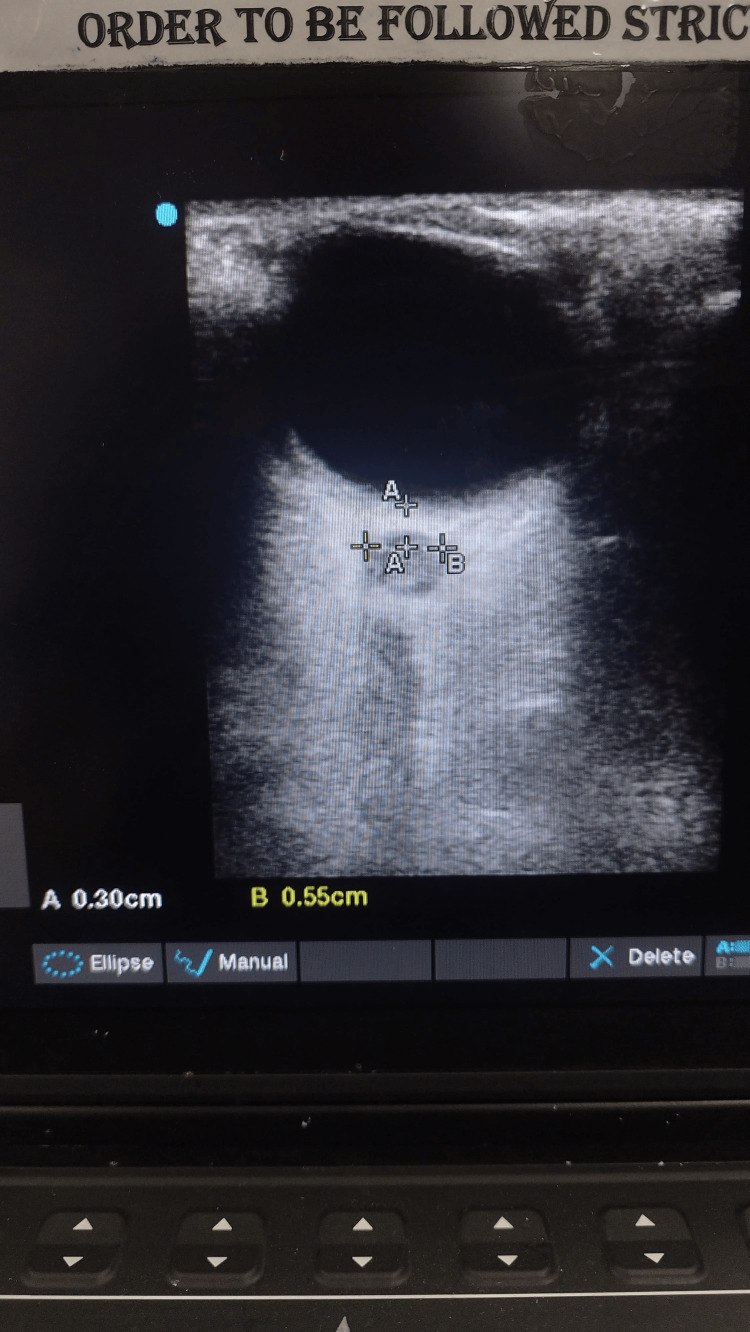
Optic Nerve Sheath Diameter (ONSD) Ultrasound The optic nerve sheath diameter (ONSD) is measured 3mm behind the retina, from which point a transverse line is drawn from the inner edge to the inner edge of both vertical hypoechoic lines.

ONSD was measured by the study investigator, who had been trained for two months under the supervision of a radiologist. The 10 MHz linear probe used in this study (SONOSITE M-Turbo, Fujifilm Sonosite India Private Limited, India) offers an axial resolution of approximately 0.1 mm and a lateral resolution of around 0.2 mm at the depth of the retrobulbar optic nerve, making it suitable for accurate measurement of the optic nerve sheath diameter [12]. The patient was placed in a supine position with their head in a neutral position (15-30° elevation), and sterile ultrasonic gel was applied to the closed eyelid. Beneath an anechoic globe, the optic nerve sheath is visible as a hypoechoic band. Using accepted methods outlined in the literature, the transorbital optic nerve sheath diameter of each side was measured and then modified to obtain orbital sonography. An axial cross-sectional picture of the optic nerve was obtained by positioning the probe in both transverse and longitudinal orientations. The distance measured was 0.3 cm from the back of the optic disc (Figure 1). The average of both the measurements was calculated, and then the average of the right and left was used. Measurements were taken on the day of admission and on days one and three.

Data collection

**Figure 2:**
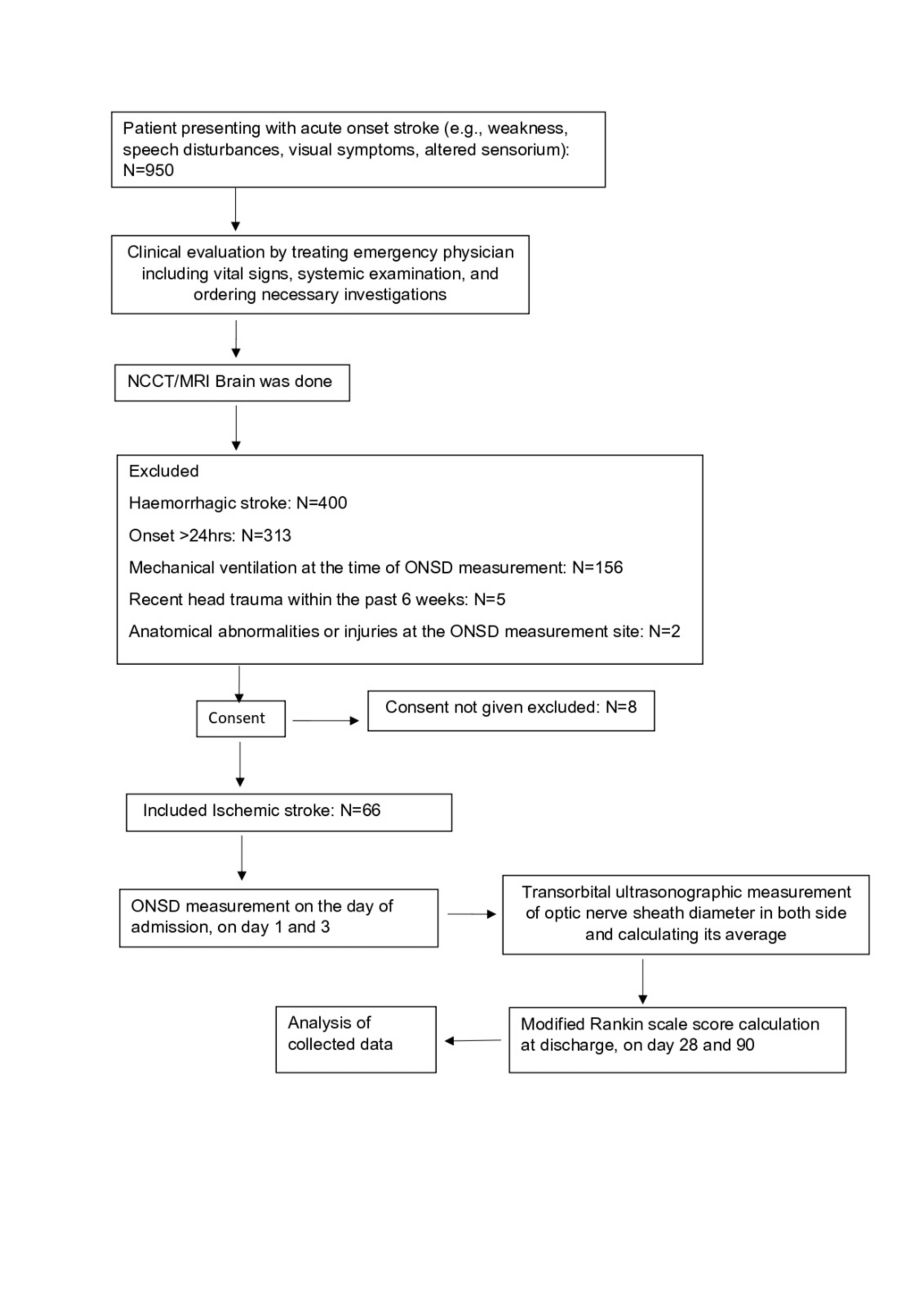
Flow Chart NCCT: non contrast computed tomography, MRI: magnetic resonance imaging, ONSD: optic nerve sheath diameter.

Patients were followed for the duration of hospital stay, and functional outcome in terms of mRS score was collected on day 28 and day 90 via telephone for subjects who survived to discharge. The assessor was blinded to the patient's ONSD measurements and clinical imaging findings to minimize outcome assessment bias. The study flow chart is attached (Figure 2).

Outcomes

Using the mRS score, the primary outcome was the functional outcome at 28 and 90 days. On the mRS, a score of 0-3 indicated good functional results (no symptoms to mild disability), while a score of 4-6 indicated poor functional outcomes (moderate disability to death). The secondary outcome was mortality at 28 and 90 days. Patients who did not survive on day 28 and 90 were considered as mRS score 6 [13]. 

Statistical analyses

For the continuous variables, descriptive statistics such as mean [standard deviation (SD)] and median [interquartile range (IQR)] were used. Frequencies and percentages for categorical variables were computed; the association between two categorical variables was explored using the chi-square test. Change in ONSD over time within each group was analysed using the Friedman test. When the data was not normally distributed, the Wilcoxon-Mann-Whitney Test was used to compare the ONSD in two groups, and when the data was normally distributed, parametric tests (t-test) were used to compare the groups. The average ONSD (cm) of the two groups was compared for overall change over time using the generalised estimating equations approach. Post-hoc pairwise analysis was performed to explore the difference in ONSD at a particular time point from the day 0 time point. The ROC curves were plotted for each of the three ONSD difference scores, and the areas under the curves were analysed. Predictor is ONSD difference scores, response is mRS score (Dichotomized) on day 28 and day 90 (scores less than or equal to 3 - good; scores more than 3- bad). The data was entered in an Excel sheet and analysed with the help of SPSS software version 23 (IBM Corporation, Armonk, New York).

## Results

Sixty-six individuals who had suffered an acute ischemic stroke were included in the study over the research period. Mean age was 55.30 ± 13.99, and 56.1% of individuals were male. The average ONSD (cm) was 0.39 (0.05), 0.39 (0.05), and 0.39 (0.05) on all measurement days: day 0, one, and three in the good functional outcome group. The mean ONSD was 0.54 (0.10), 0.54 (0.10), and 0.56 (0.11) on day 0, one, and three, respectively, in the poor functional outcome group on day 90. It was substantially higher in the poor functional outcome group than in the good outcome group at all measuring time points (day 0, one, and three). In the good functional outcome group, change over time in ONSD was not statistically significant from day 0 to day three (Friedman Test: χ2 = 0.2, p = 0.897). In the poor functional outcome group, change over time in ONSD was statistically significant from day 0 to day three (Friedman Test: χ2 = 27.3, p < 0.001) (Table 1). Forty-four (66.7%) participants had a poor functional outcome, and one (1.5%) participant had mortality on day 28. However, on day 90, 37 (56.1%) participants had a poor functional outcome, and 20 (30.8%) participants had mortality.

**Table 1:** Change in Average ONSD (cm) Over Time Within Each Group, Comparison Between Two Groups (Good and Poor Functional Outcome at 90 Days) at All-Time Points and Overall Comparison SD: standard deviation

Average ONSD (cm)	Functional Outcome (Day 90)		p value for comparison of the two groups at each of the time points (Wilcoxon-Mann-Whitney Test)
	Good	Poor	
	Mean (SD)	Mean (SD)	
Day 0	0.39 (0.05)	0.54 (0.10)	< 0.001
Day 1	0.39 (0.05)	0.54 (0.10)	< 0.001
Day 3	0.39 (0.05)	0.56 (0.11)	< 0.001
p value for change in Average ONSD (cm) over time within each group (Friedman Test)	0.897	< 0.001	
Overall p value for comparison of change in Average ONSD (cm) over time between the two groups (Generalized Estimating Equations)	< 0.001		

The Wilcoxon-Mann-Whitney test was used to compare the two groups in terms of average ONSD (cm) at each of the time points. The two groups differed significantly in terms of average ONSD (cm) at the following time points: day 0, day one, and day three. The maximum change from the day 0 time point was observed at the day three time point (Table 2).

**Table 2:** Association Between Functional Outcome (Day 28 & 90) and Average ONSD (cm)

ONSD Median (Average) (cm)	Outcome (Day 28)		Wilcoxon-Mann-Whitney U Test		Outcome (Day 90)		Wilcoxon-Mann-Whitney U Test	
	Good	Poor	W	p value	Good	Poor	W	p value
Day 0	0.37 (0.36-0.44)	0.56 (0.44-0.6)	160.500	< 0.001	0.37 (0.36-0.44)	0.56 (0.46-0.61)	119.500	< 0.001
Day 1	0.36 (0.36-0.45)	0.55 (0.44-0.59)	-6.423 (t-test)	< 0.001	0.37 (0.36-0.45)	0.56 (0.46-0.61)	126.000	< 0.001
Day 3	0.37 (0.35-0.44)	0.56 (0.44-0.64)	-6.480 (t-test)	< 0.001	0.37 (0.35-0.45)	0.59 (0.47-0.66)	132.500	< 0.001

**Figure 3:**
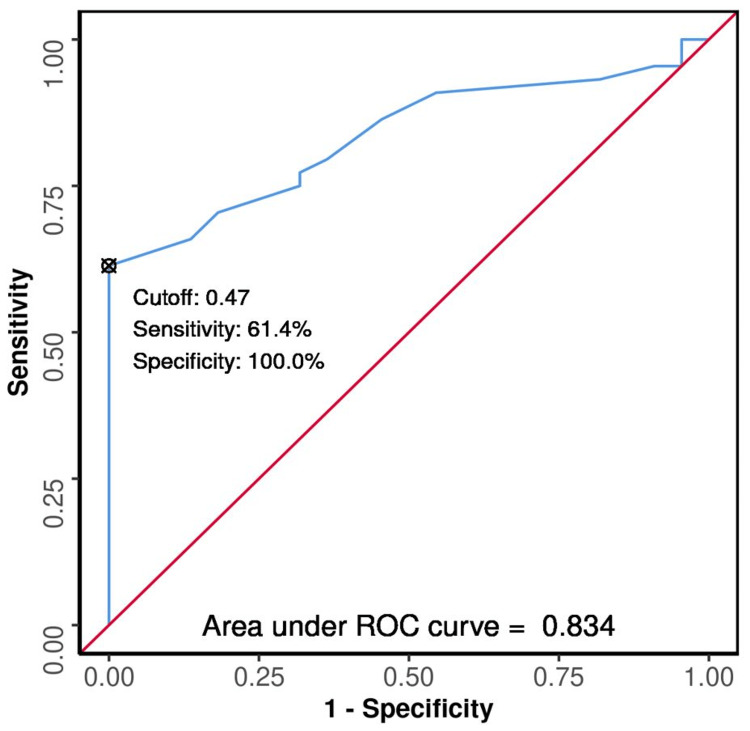
ROC Curve Analysis Showing Diagnostic Performance of Average ONSD (cm) Day 0 in Predicting Functional Outcome on Day 28 ROC: receiver operating characteristic; ONSD: optic nerve sheath diameter

**Figure 4:**
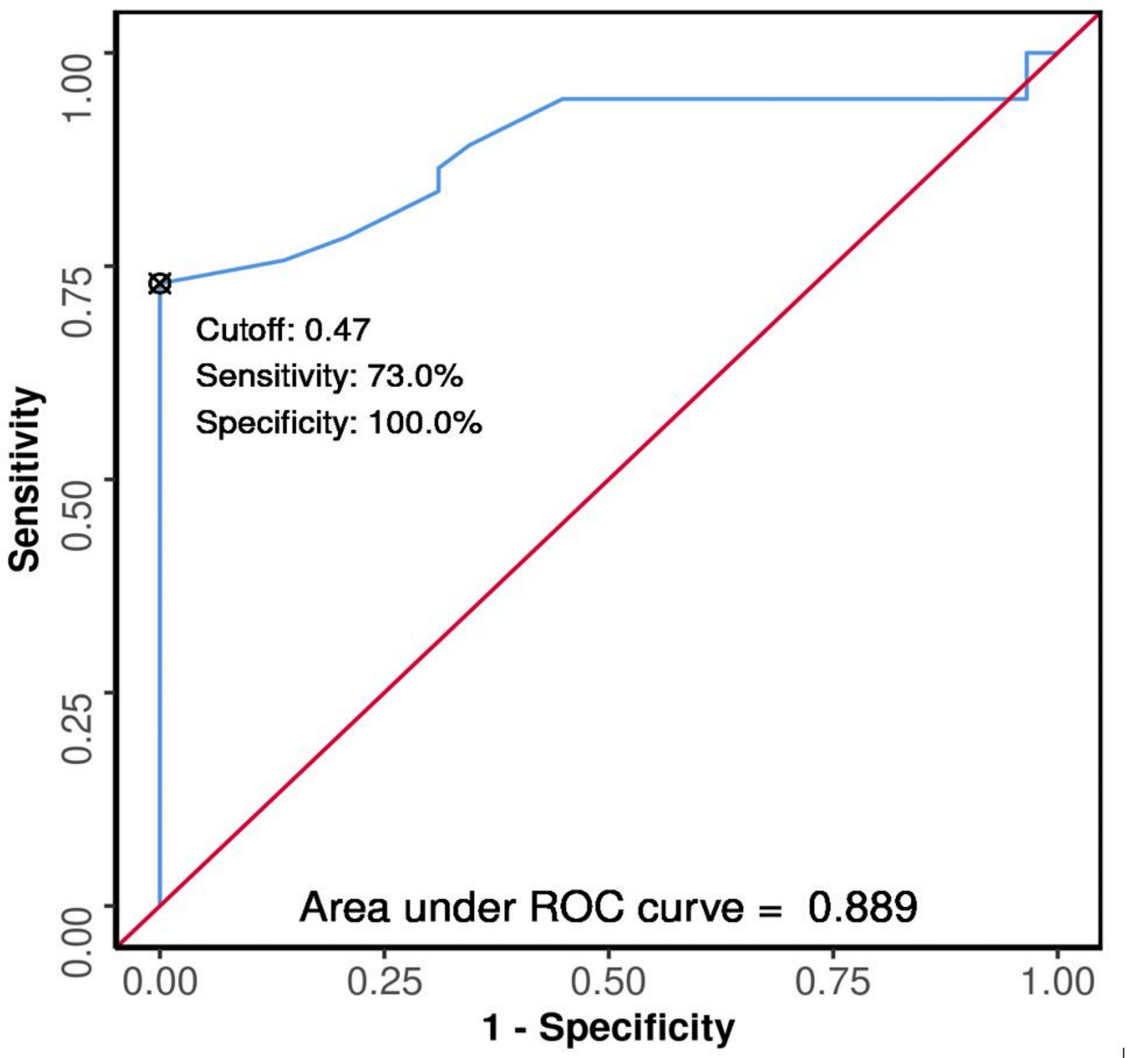
ROC Curve Analysis Showing Diagnostic Performance of Average ONSD (cm) Day 0 in Predicting Functional Outcome on Day 90 ROC: receiver operating characteristic; ONSD: optic nerve sheath diameter

**Figure 5:**
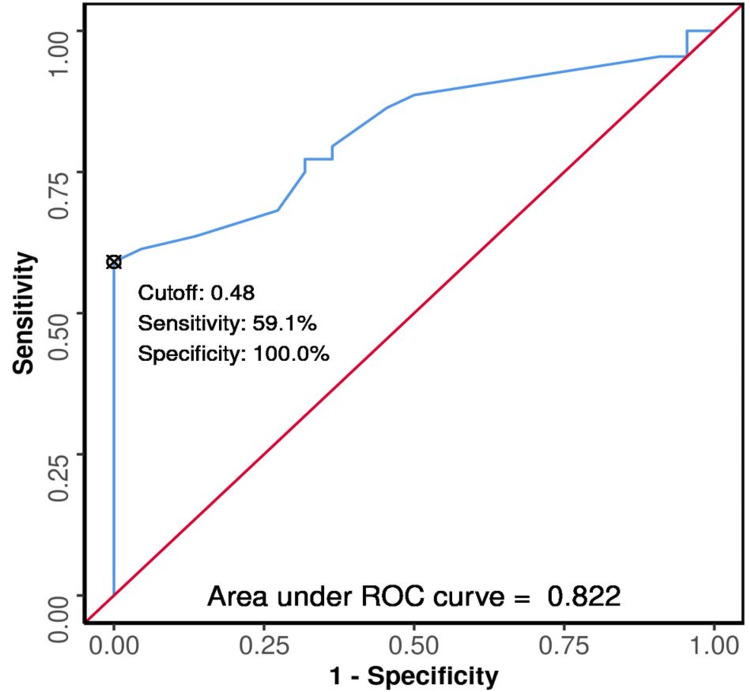
ROC Curve Analysis Showing Diagnostic Performance of Average ONSD (cm) Day 1 in Predicting Functional Outcome on Day 28 ROC: receiver operating characteristic; ONSD: optic nerve sheath diameter

**Figure 6:**
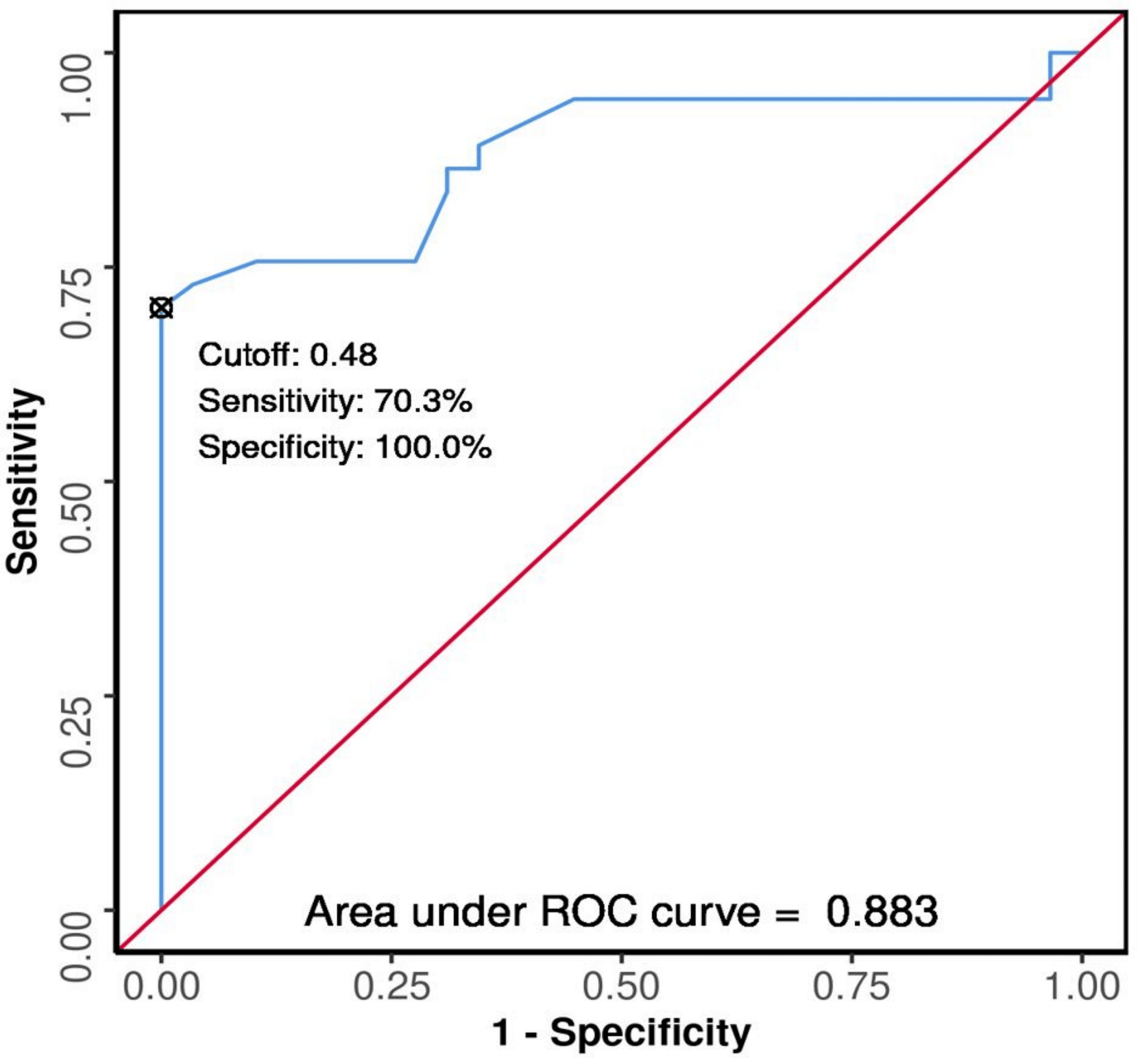
ROC Curve Analysis Showing Diagnostic Performance of Average ONSD (cm) Day 1 in Predicting Functional Outcome on Day 90 ROC: receiver operating characteristic; ONSD: optic nerve sheath diameter

**Figure 7:**
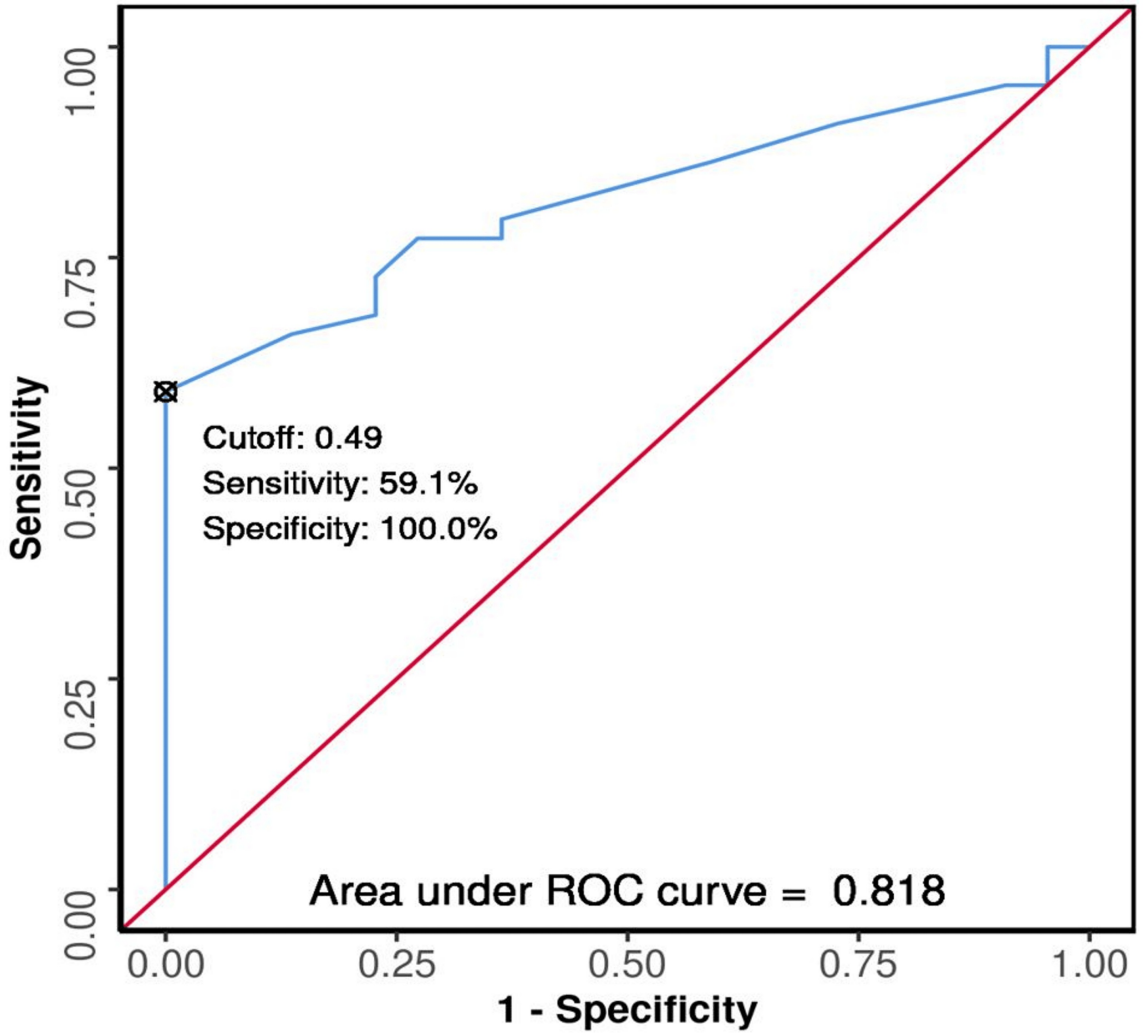
ROC Curve Analysis Showing Diagnostic Performance of Average ONSD (cm) Day 3 in Predicting Functional Outcome on Day 28 ROC: receiver operating characteristic; ONSD: optic nerve sheath diameter

**Figure 8:**
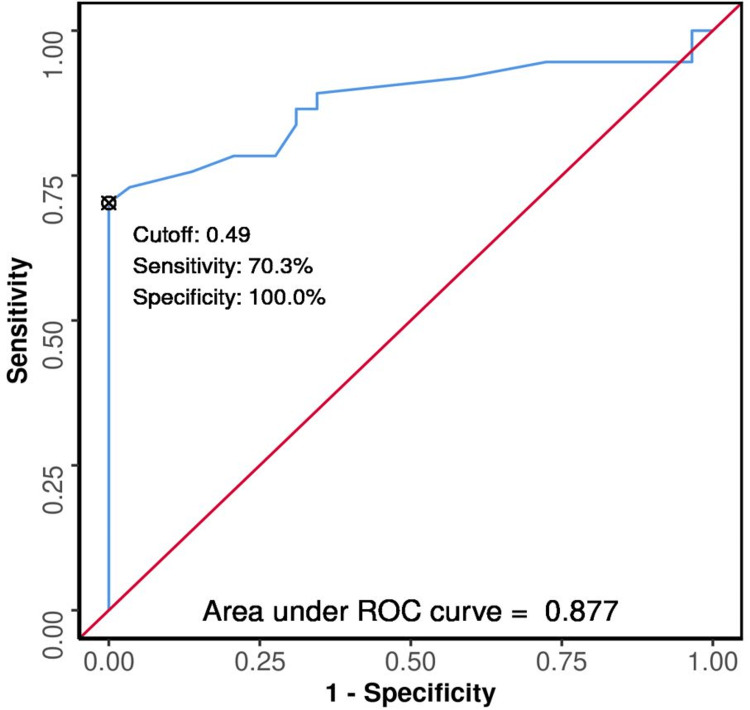
ROC Curve Analysis Showing Diagnostic Performance of Average ONSD (cm) Day 3 in Predicting Functional Outcome on Day 90 ROC: receiver operating characteristic; ONSD: optic nerve sheath diameter

The ROC curves were plotted (Figures 3, 4, 5, 6, 7, 8) to see the area under curve (AUROC) and to find a cut-off value with the ability to predict poor functional outcome on day 28 and 90 (Figures 3, 4, 5, 6, 7, 8).

The area under the ROC curve (AUROC) for average ONSD (cm) on day 0 in predicting poor vs good outcome on day 28 was 0.834 [95% confidence interval (CI): 0.739 - 0.929]; similarly, on day 90, it was 0.889 (95% CI: 0.805 - 0.972), thus demonstrating good diagnostic performance. It was statistically significant (p < 0.001). At a cutoff of average ONSD (cm) (day 0) ≥ 0.47, it predicts poor functional outcome (day 28 and 90) with a sensitivity of 61% and 73%, and a specificity of 100%.

The area under the ROC curve (AUROC) for average ONSD (cm) on day one in predicting poor vs good outcome on day 28 was 0.822 (95% CI: 0.724 - 0.92), similarly on day 90 it was 0.883 (95% CI: 0.797 - 0.968), thus demonstrating good diagnostic performance. It was statistically significant (p < 0.001). At a cutoff of Average ONSD (cm) (day 1) ≥ 0.48, it predicts poor functional outcome (day 28 and 90) with a sensitivity of 59% and 70%, and a specificity of 100%.

The area under the ROC curve (AUROC) for average ONSD (cm) on day three in predicting poor vs good outcome on day 28 was 0.818 (95% CI: 0.719 - 0.917), similarly, on day 90 it was 0.877 (95% CI: 0.789 - 0.964), thus demonstrating good diagnostic performance. It was statistically significant (p < 0.001). At a cutoff of average ONSD (cm) on day three, a value of ≥ 0.49, it predicts Poor functional outcome (day 28 and 90) with a sensitivity of 59% and 70%, and a specificity of 100%.

Statistical comparison with DeLong's Test did not show any significant difference in diagnostic accuracy of ONSD on other days, i.e., day one and day three.

Strength of association (point-biserial correlation) between average ONSD on day three with poor functional outcome on day 90 was 0.68. The diagnostic accuracy to predict poor functional outcome on day 90 is highest for ONSD on day 0. Statistical comparison with DeLong's test did not show any significant difference in diagnostic accuracy of ONSD on other days, i.e., day 0, day one, and day three (Tables 3, 4).

**Table 3:** Comparison of the Diagnostic Performance of ONSD in Predicting Poor Functional Outcome on Day 28 and 90 (Description of Variables) ONSD: optic nerve sheath diameter

Day 28	ONSD (Average) (cm)	Poor outcome	Good outcome	Total Positives	True Positives	True Negatives	False Positives	False Negatives
	Day 0 (Cutoff: 0.47 by ROC)	≥ 0.47	< 0.47	27 (40.9%)	27 (41%)	22 (33%)	0 (0%)	17 (26%)
	Day 1 (Cutoff: 0.48 by ROC	≥ 0.48	< 0.48	26 (39.4%)	26 (39%)	22 (33%)	0 (0%)	18 (27%)
	Day 3 (Cutoff: 0.49 by ROC)	≥ 0.49	< 0.49	26 (39.4%)	26 (39%)	22 (33%)	0 (0%)	18 (27%)
Day 90	Day 0 (Cutoff: 0.47 by ROC)	≥ 0.47	< 0.47	27 (40.9%)	27 (41%)	29 (44%)	0 (0%)	10 (15%)
	Day 1 (Cutoff: 0.48 by ROC	≥ 0.48	< 0.48	26 (39.4%)	26 (39%)	29 (44%)	0 (0%)	11 (17%)
	Day 3 (Cutoff: 0.49 by ROC)	≥ 0.49	< 0.49	26 (39.4%)	26 (39%)	29 (44%)	0 (0%)	11 (17%)

**Table 4:** Primary Diagnostic Parameters

Day 28	ONSD (Average) (cm)	Sn	Sp	PPV	NPV	P Value	DA
	Day 0 (Cutoff: 0.47 by ROC)	61.4% (45-76)	100.0% (85-100)	100.0% (87-100)	56.4% (40-72)	< 0.001	74.2% (62-84)
	Day 1 (Cutoff: 0.48 by ROC	59.1% (43-74)	100.0% (85-100)	100.0% (87-100)	55.0% (38-71)	< 0.001	72.7% (60-83)
	Day 3 (Cutoff: 0.49 by ROC)	59.1% (43-74)	100.0% (85-100)	100.0% (87-100)	55.0% (38-71)	< 0.001	72.7% (60-83)
Day 90	Day 0 (Cutoff: 0.47 by ROC)	73.0% (56-86)	100.0% (88-100)	100.0% (87-100)	74.4% (58-87)	< 0.001	84.8% (74-92)
	Day 1 (Cutoff: 0.48 by ROC	70.3% (53-84)	100.0% (88-100)	100.0% (87-100)	72.5% (56-85)	< 0.001	83.3% (72-91)
	Day 3 (Cutoff: 0.49 by ROC)	70.3% (53-84)	100.0% (88-100)	100.0% (87-100)	72.5% (56-85)	< 0.001	83.3% (72-91)

## Discussion

According to the findings of our investigation, serial measurement of ONSD by ultrasonographic examination may be useful in predicting a poor functional prognosis for patients with acute ischaemic stroke who present to the emergency room. The optic nerve sheath is continuous with the subarachnoid space of the brain; hence, a rise in ICP due to any cause will be reflected as an increase in the diameter of the optic nerve sheath. It usually appears within one to five days of raised ICP, even more rapidly (within two to eight hours) in case of subarachnoid haemorrhage [14]. Dubourg et al. conducted a systematic review and meta-analysis to determine the diagnostic accuracy of ultrasonography of optic nerve sheath diameter to detect raised ICP, which included six studies and 231 patients, and demonstrated a high level of accuracy in diagnosing intracranial hypertension. The pooled sensitivity for detection of raised intracranial pressure was 0.90 (95% CI 0.80 to 0.95; six studies), the pooled specificity was 0.85 (95% CI 0.73 to 0.93; six studies), and the pooled diagnostic odds ratio was 51 (95% CI 22 to 121). The summary ROC curve area was 0.94 (95% CI 0.91 to 0.96). The positive likelihood ratio was 6.11 (95% CI 3.28 to 11.37), and the negative likelihood ratio was 0.12 (95% CI 0.06 to 0.23). None of the studies revealed any evidence of statistical heterogeneity that was particularly significant. In adult patients with traumatic brain injury and intracranial haemorrhage, they concluded that ultrasonography of the optic nerve sheath diameter demonstrated a good level of diagnostic accuracy for identifying intracranial hypertension [15].​ In 2025, Danış et al. conducted a prospective observational study that evaluated the role of bedside ultrasonographic ONSD measurement in diagnosing hypertensive emergency and predicting hospitalization in patients with hypertensive crisis. Among 112 patients, those with hypertensive emergency had significantly higher ONSD values (5.99 ± 0.65 mm) than those with hypertensive urgency (5.11 ± 0.57 mm, p < .001). Hospitalized patients also showed elevated ONSD levels. An ONSD cutoff of 5.8 mm demonstrated good diagnostic performance (sensitivity 68%, specificity 93.1%). ONSD measurement is a simple, noninvasive tool that may support early diagnosis and management decisions in the ED [16]. Seyedhosseini et al. conducted a study in 2017 with 60 patients with acute stroke presentations (over six months). The relationship between mean ONSD and study variables was investigated. The mean ± SD ONSD in the deceased cases was 4.40 ± 0.64 mm, while it was 3.83 ± 0.56 mm in the survivors. The Youden index determined that ONSD > 3.9 mm was the best cut-off point for mortality prognosis. It has an 83.3% sensitivity and a 59.2% specificity [17].​​​

Only a few studies have been conducted to analyse the use of serial ONSD measurements in stroke individuals. There is only one study on serial ONSD assessment in ischemic stroke patients. Patients with middle cerebral artery (MCA) infarction who are at risk of developing malignant MCA syndrome can benefit from daily ONSD monitoring [18]. A study conducted by Dias et al. in 2017, enrolling 44 patients with acute intracerebral hemorrhage in whom ONSD was measured ultrasonographically, concluded that increased ONSD is an independent predictor of mortality at three months [19].

On the other hand, Strumwasser et al. conducted a blinded prospective analysis of adult trauma patients who needed ICP monitoring. Ultrasound was used to measure the ONSD both before and after an ICP monitor was implanted (Camino Bolt or Ventriculostomy). The particular objectives were to assess ONSD's precision in estimating increased ICP; compare ONSD and ICP in unilateral and bilateral head traumas. They concluded that sonographic ONSD has poor accuracy and correlation, and it is unreliable as a substitute for increased ICP in place of intrusive monitoring [20].

Optic nerve sheath diameter

The mean ONSD values recorded on day 0, day 1, and day 3 in our study remained relatively stable, averaging around 0.48 cm. While these values are consistent with the upper end of the normal range reported in healthy individuals, they are slightly lower than those observed in other ischemic stroke populations. For example, Manouchehrifar et al. reported a mean ONSD of 5.5 ± 0.4 mm in ischemic stroke patients. The discrepancy may be attributable to differences in measurement techniques (e.g., probe frequency, angle of insonation), patient demographics, stroke severity, or timing of assessment. Our protocol measured ONSD within the first 72 hours, which may have captured patients before the maximal intracranial pressure rise occurred in some cases. These variations highlight the importance of standardizing sonographic technique and timing when using ONSD as a prognostic marker in stroke [21]. In 2017, Gökcen et al. compared ONSD between a control group and those suffering from cerebrovascular illness. They found that ONSD was considerably higher in the case group, particularly when there was anterior circulation involvement (p < 0.001). This variation in ONSD measurements in various studies could be attributable to a variety of factors, encompassing genetic and cultural variety, ultrasound method measurement, and interobserver variability [22].

Change in ONSD over time in good and poor functional outcome groups

Patients with an mRS score ≤ 3 on day 28, 90 were grouped in the good functional outcome group, and those having a higher mRS score were grouped as the poor functional outcome group. Subjects with poor functional outcome had a significantly higher ONSD (average 0.54 cm) at all time points, compared with good functional outcome (average mean 0.39 cm) ischemic stroke subjects. In good functional outcome patients on day 28 and day 90, the change in ONSD over time was not statistically significant from day 3 - day 0 and but it was statistically significant in poor functional outcome patients.

Change in optic nerve sheath diameter over three days in all ischemic stroke patients

Change in ONSD (day one to day 0) was compared with change in ONSD (day three to day 0); the change was found to be significant on the third day compared to day one. In the first 24 hours following an ischaemic stroke, brain oedema is typically not a major concern, except for bigger cerebellar infarctions, as per a study by Dostovic et al. [9]. It begins to manifest 24 to 48 hours after the onset of acute ischaemic stroke and peaks three to five days later. It was observed that the difference between the ONSD on day three and day 0 is greater than the same on day one and day 0. Thus, in cases of acute ischaemic stroke, measuring the ONSD on days 0 and three can aid in predicting the functional prognosis. According to our study, following trends of ONSD makes prediction better, i.e., an increase in ONSD over the third day can predict a poor functional outcome of the 90th day. Patel et al. found that the odds ratio for death among ischaemic stroke patients increased by 4.2 for every 0.1 cm increase in ONSD [23].

Limitations of the study

Although our study had an adequate sample size to support statistical analysis, the overall number of participants was relatively modest. Larger multicenter studies are needed to validate and strengthen the generalizability of our findings.

In this study, ONSD was measured exclusively using ultrasonography. While this method is convenient and non-invasive, we did not compare these measurements with those obtained via CT or MRI. Correlating ONSD with imaging-based markers of intracranial pressure-such as midline shift or cerebral edema-could enhance diagnostic accuracy and confirm the anatomical basis of elevated ONSD.

Additionally, although all patients were managed according to standard acute stroke treatment protocols, individual-level treatment data (e.g., thrombolysis, endovascular therapy, decompressive surgery) were not captured. These interventions can significantly influence patient outcomes and may act as confounding variables. Future research should incorporate both baseline stroke severity assessments (e.g., NIHSS scores) and detailed treatment data to better model the relationship between ONSD and clinical outcomes.

A further limitation is that all ONSD measurements were performed by a single operator. While this reduces inter-operator variability within the study, it limits reproducibility and external validity. Ultrasound-based ONSD assessments are known to be operator-dependent, and the lack of inter-observer reliability analysis is a notable constraint.

Moreover, outcome assessment via the mRS was conducted through telephone follow-up, which may have introduced recall or interviewer bias. In-person evaluations might have yielded more reliable functional assessments.

Finally, although we observed that day three ONSD values were associated with poor outcomes, we did not perform a reclassification analysis to assess how many patients crossed the diagnostic threshold between day 0 and day three. Such dynamic changes could offer further insight into the prognostic utility of serial ONSD monitoring.

## Conclusions

We discovered that a larger optic nerve sheath width in acute ischaemic stroke patients presenting to the emergency department was associated with poor functional outcomes judged by an mRS score of more than 3 at 28, 90 days. ONSD ≥ 0.47cm on day 0 predicts a relatively bad functional result (mRS > 3) on day 90. This study demonstrates that increased optic nerve sheath diameter (ONSD) measured via bedside ultrasonography is associated with poor functional outcomes in patients with acute ischemic stroke. ONSD assessment is a non-invasive, easily repeatable tool that may aid in early risk stratification and prognostication. Measurement of ONSD can predict functional outcome on day 90 better than that on day 28. Specificity of the study for predicting outcome is satisfactory; however sensitivity of predicting outcome on days 28 and 90 is limited.
